# Role of Hydrogen in Ethylene-Based Synthesis of Single-Walled Carbon Nanotubes

**DOI:** 10.3390/nano13091504

**Published:** 2023-04-28

**Authors:** Alisa R. Bogdanova, Dmitry V. Krasnikov, Eldar M. Khabushev, Javier A. Ramirez B., Yakov E. Matyushkin, Albert G. Nasibulin

**Affiliations:** 1 Skolkovo Institute of Science and Technology, Nobelya Str. 3, Moscow 121205, Russiad.krasnikov@skol.tech (D.V.K.); javier.ramirez@skoltech.ru (J.A.R.B.); 2 Moscow Institute of Physics and Technology, Institute Lane 9, Dolgoprudny 141701, Russia

**Keywords:** single-walled carbon nanotubes, chemical vapor deposition, hydrogen, ethylene, pyrolysis

## Abstract

We examined the effect of hydrogen on the growth of single-walled carbon nanotubes in the aerosol (a specific case of the floating catalyst) chemical vapor deposition process using ethylene as a carbon source and ferrocene as a precursor for a Fe-based catalyst. With a comprehensive set of physical methods (UV-vis-NIR and Raman spectroscopies, transmission electron microscopy, scanning electron microscopy, differential mobility analysis, and four-probe sheet resistance measurements), we showed hydrogen to inhibit ethylene pyrolysis extending the window of synthesis parameters. Moreover, the detailed study at different temperatures allowed us to distinguish three different regimes for the hydrogen effect: pyrolysis suppression at low concentrations (I) followed by surface cleaning/activation promotion (II), and surface blockage/nanotube etching (III) at the highest concentrations. We believe that such a detailed study will help to reveal the complex role of hydrogen and contribute toward the synthesis of single-walled carbon nanotubes with detailed characteristics.

## 1. Introduction

Though carbon nanotubes do not have a clear date of discovery [1], during the last 30 years, researchers have invested enormous efforts to pursue novel physical effects or next-generation devices based on nanotubes. Being a family of materials with a wide range of properties, carbon nanotubes, as a rule, are distinguished by the number of layers [2]. Multi-walled carbon nanotubes usually show a lower price, higher defectiveness, and the ability to sacrifice the outer layer for functionalization. Multi-walled carbon nanotubes show promising results in composites, energetics, and functional materials, while single-walled carbon nanotubes (SWCNTs) provide higher prospects in electronics, optics, and medicine [3,4,5,6,7]. However, sometimes SWCNTs also contribute to the polymer composites [8,9] owing to the significant drop in price induced by the OCSiAl company [10,11].

SWCNTs proved to be one of the most promising materials for various applications due to their unique ensemble of optical, electronic, and mechanical properties [12,13,14]. A combination of high optical transparency, intrinsic conductivity, and flexibility makes SWCNT-based thin films an ideal candidate for transparent electrodes [15,16]. Since SWCNT properties strongly depend on their structure [17], precise control over their characteristics is the key barrier to their successful industrial applications. Among different techniques for carbon nanotube growth, the aerosol chemical vapor deposition (CVD) method is one of the most suitable for advanced control over individual nanotube characteristics, such as diameter, chirality, and length [18]. However, the entangled relationship between the growth conditions/reactor parameters and SWCNT characteristics inhibits the creation of a universal model for the process to reach the tailored properties. This results in the active development of phenomenological methods (e.g., machine learning) or extensive data analysis [19,20,21].

Another opportunity is to boost the nanotube growth indirectly using, for example, so-called promotors: species added in small portions to enhance a specific parameter. Such promoters of the nanotube growth as water, potassium, sulfur, and carbon dioxide proved themselves as additives, enhancing the yield of synthesis, etching amorphous carbon from the surface of the catalyst, and increasing nanotube diameters [22,23,24,25,26]. Usually formed during growth or introduced as a carrier gas, hydrogen is another indirect booster whose role is not fully understood yet. Some researchers report the phenomenological effect on the SWCNT growth and yield enhancement accompanied by defectiveness increase [27]. On the contrary, others claim that labile hydrogen is unfavorable for growth due to excessive etching [28].

Moreover, some observe several regimes of the hydrogen effect on the synthesis [29,30,31,32,33,34]. Rao et al. [30] showed a low hydrogen concentration to increase the yield of SWCNTs from methane as deactivation induced by excessive carbon species on the catalyst surface was inhibited, and more particles remained active. Nevertheless, when the hydrogen to methane ratio exceeded unity at 1000 °C, carbon feedstock to the catalyst suffered from inhibited methane decomposition, reducing the yield of SWCNTs. At the same time, Li et al. [32] observed 15–35% of hydrogen (C_2_H_4_ as a carbon source at 750 °C) to improve the SWCNT quality for substrate CVD growth of vertically aligned carbon nanotubes. Ma et al. comprehensively studied the formation of multi-walled carbon nanotubes on quartz and alumina revealing not only the complex role of hydrogen (promotion at low amounts and carbon gasification to methane at high) but also the substrate effect [33]. Zhang et al. also highlighted the complex relationship between the carbon source and hydrogen [34]. Moreover, the authors showed that the excess hydrogen [35] changed the crystal phase of the catalyst from Fe_3_C into body-centered cubic Fe, leading to the formation of more sp^3^ structure defects by the saturated carbon atoms. Eventually, under suitable hydrogen concentration (15%), it etched the catalyst to form evenly distributed iron particles, maximizing catalytic efficiency and reducing the formation of amorphous carbon. In addition, hydrogen was long known to inhibit pyrolytic carbon formation [36], especially in the case of methane pyrolysis [36,37,38,39]. Hydrogen inhibits active radicals and terminates the chain, thereby stopping pyrolysis and resulting in smaller carbon crystallites [40]. Thus, studies revealed the following hydrogen effect on carbon nanotube synthesis: hydrogen can help to prolong the catalyst lifetime by etching the impurities on its surface [29,30,31,32] and shifts the crystal phase of the catalyst if in excess [32]. However, many questions about the hydrogen influence remain with no answers. Here, we wish to contribute to the understanding of the hydrogen role during the aerosol chemical vapor deposition (CVD)—specific case of the floating catalyst process under extreme dilution, as the method provides state-of-the-art carbon nanotubes for optoelectronics [15].

In this work, to reduce the complexity of the effect, we employed aerosol CVD with a single carbon source—ethylene, thereby removing the effect of a substrate or multiple hydrocarbons. We examine the effect of hydrogen on the growth of SWCNTs produced employing the aerosol CVD method, using ethylene as a carbon source and ferrocene as a catalyst precursor. Using a comprehensive set of methods (aerosol spectrometry, SEM, TEM, UV-vis-NIR spectroscopy, Raman spectroscopy, and four-point probe measurements), we thoroughly investigate the role of hydrogen on carbon nanotube growth, assessing the yield, diameter distribution, length distribution, aerosol concentration, quality, and sheet resistance. We observe three distinct SWCNT growth regimes depending on the hydrogen concentration. These findings help to understand the ability of hydrogen to tune the characteristics and purity of the produced SWCNTs.

## 2. Materials and Methods

**Figure 1:**
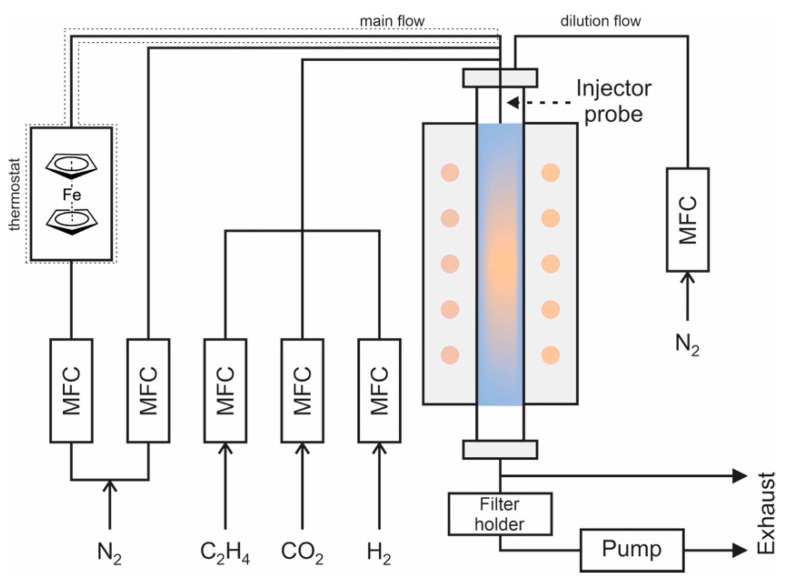
Scheme of the reactor for the aerosol CVD synthesis.

Single-walled carbon nanotubes were produced using the aerosol chemical vapor deposition method (CVD)—the specific case of floating catalyst CVD with an extreme dilution of catalyst particles. The aerosol CVD synthesis reactor (Figure 1) [41] consisted of a tubular glass with an inner diameter of 51 mm inserted inside the three-zone furnace with a length of 1300 mm (isothermal hot zone ~550 mm). Ferrocene vapor (the precursor of Fe-based catalyst, Fe(C_5_H_5_)_2_, 98%, Sigma Aldrich) was transferred from a ferrocene cartridge by nitrogen gas flow (99.999%) to the reactor. The partial vapor pressure of ferrocene was 0.28 Pa during all the experiments. The catalyst-containing flow was fed through the injector near the hot zone of the reactor and mixed with ethylene (carbon source 0–0.03 lpm, 99.9%), CO_2_ (0–0.05 lpm, 99.995%), and H_2_ (0–0.6 lpm, 99.999%), reaching the total flow of 2.5 lpm (this parameter remained constant throughout the experiments). The temperature was in the range of 900–1000 °C. The aerosol of the synthesized SWCNTs was filtered with a nitrocellulose filter (HAWP, Merck Millipore) with a typical collection time of ca. 1.5 min for further dry transfer to a glass substrate [42].

It should be noted that unlike numerous advantages of the aerosol CVD method (control of bundling, facile methods for catalyst formation, absence of catalyst substrates), it is limited in terms of direct observation of catalyst/nanotube within the process or in situ simulation (due to a complex and non-isothermal evolution the catalyst goes through). Thus, we employed a set of methods to discuss the trends in nanotube features as fingerprints of changes in the catalytic process. As the transparent electrodes are one of the main applications of aerosol CVD SWCNT films, we referred to key performance parameters of this field to increase the relevance of the work; i.e., we employed wavelength 550 nm—the middle of the visible range—for transmittance and equivalent sheet resistance as a thickness-independent parameter to compare different films [15,43,44].

As aerosol CVD setup produces not SWCNT powders, but thin films with controlled transparency, we discuss the reactor productivity with the respect to the area of the product collected. PerkinElmer UV-vis-NIR spectrophotometer Lambda 1050 was employed to estimate the mean diameter and thickness of SWCNT film. The spectra were collected within the range of 250–2600 nm with a resolution of 2 nm. According to the Beer–Lambert law, we can associate the film thickness with absorbance at the wavelength of 550 nm; thus, the yield can be defined as the collection area of the SWCNT film with the transmittance of 90% (at a wavelength of 550 nm) from 1 L of the flow passed through the filter and calculated as:

(1) $$Yield\left[{cm}^{2}\cdot L^{-1}\right]=\frac{\log_{0.9}\left(T_{550}\right)}{t\left[min\right]\cdot Q\left[slpm\right]}\cdot \frac{\pi {\left(d\left[cm\right]\right)}^{2}}{4},$$

where *Q* is the flow rate, *d* is the diameter of the collecting area of the filter [45]. The position of the S_11_, S_22_, and M_11_ peaks associated with van Hove singularities provides an estimation for the mean diameter of SWCNTs in the film.

The sheet resistance of the SWCNT films was measured with a four-probe station Jandel RM3000 at least five times at various orientations. Depending on the sample conductivity, we varied the current from 0.01 to 1 mA to operate in the 20–50 mV range (optimal for the device). The average sheet resistance value was later used to calculate equivalent sheet resistance normalized to 90% transmittance according to the following equation [15,43,44]:

(2) $$R_{90}=\frac{R_{s}A_{550}}{\log\left(10/9\right)}=R_{s}\log_{0.9}\left(T_{550}\right),$$

where $R_{s}$ is the average sheet resistance of the film, *A*_550_ is the film absorbance at a wavelength of 550 nm proportional to the film thickness [46] ($A_{550}=-log_{10}T$). The detailed procedure for the calculation of the error bars for the yield and equivalent sheet resistance can be found in SI.

Raman spectroscopy (Thermo scientific DXRxi Raman Imaging microscope; wavelength of 532 nm at 0.1 mW laser power) served to assess the quality of carbon nanotubes by determining the I_G_/I_D_ ratio. The resonant in-plane vibrational mode gives rise to the G band (the characteristic feature of the graphite lattice), whereas the D band (so-called disorder band [47]) corresponds to defects in the graphite lattice. To assess the length distribution of SWCNT bundles, the aerosol was deposited on a Si/SiO_2_ substrate located on a membrane filter from the gas phase and examined by scanning electron microscopy (SEM, JEOL JSM-7001F). To explore the morphology of SWCNTs, we placed the TEM lacey Cu-300 grid on the membrane filter and employed transmission electron microscopy (FEI Tecnai G2 F20). A differential mobility analyzer was employed to assess the number size distribution of the SWCNT aerosols. It was capable of measuring an aerosol particle diameter in a range of 0.7–60 nm (1 nm Scanning Mobility Particle Sizer Spectrometer, TSI, USA). It is worth noting that the data inversion assumed the spherical shape of the aerosol particles and the equilibrium distribution of charges for a certain particle size (standard software of Scanning Mobility Particle Sizer Spectrometer). This leads to the fact that we measured an electrical mobility diameter corresponding to an effective size of SWCNTs or their bundles in the range of 40–60 nm.

## 3. Results

### 3.1. Role of Ethyelene and Ferrocene

**Figure 2:**
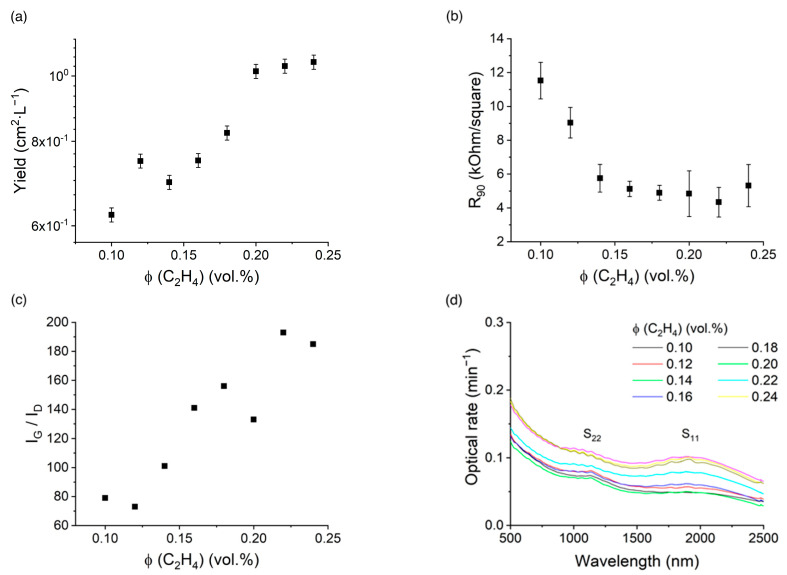
(**a**) Yield and (**b**) equivalent sheet resistance, and (**c**) Raman I_G_/I_D_ ratio as a function of carbon source concentration. (**d**) UV-Vis-NIR spectra of thin films of SWCNTs synthesized at different carbon source concentrations without adding hydrogen in the system (T = 1000 °C).

First, we investigated the influence of the ethylene concertation on the growth of SWCNTs at 1000 °C without hydrogen in the system (Figure 2). The yield increased at higher ethylene concentrations (Figure 2a), which can be naturally attributed to the rise in the carbon flux. Additionally, Figure 2b demonstrates a reversed J-shaped dependence of the equivalent sheet resistance: it declined under low ethylene concentrations and then stabilized at higher values (0.18–0.24 vol.%). Moreover, we observed that the highest yield (1.05 cm^2^∙L^−1^) was achieved at *ϕ* (C_2_H_4_) = 0.24 vol.%, corresponding to the lowest equivalent sheet resistance within the set (R_90_ ≈ 5 kOhm/sq). Recently, Khabushev et al. showed that the yield of single-walled carbon nanotubes in aerosol CVD is mostly determined by the length of SWCNTs below 925 °C, while the catalyst activation degree plays a crucial role at higher temperatures [48]. The length of carbon nanotubes is inversely proportional to the equivalent sheet resistance [49]. Thus, in our case, we considered the ethylene introduction to increase the nanotube length, while the moderate increase in the equivalent sheet resistance can be associated with higher absorbance of the films due to the formation and deposition of the polyaromatic hydrocarbons on the surface of the SWCNTs [50].

**Figure 3:**
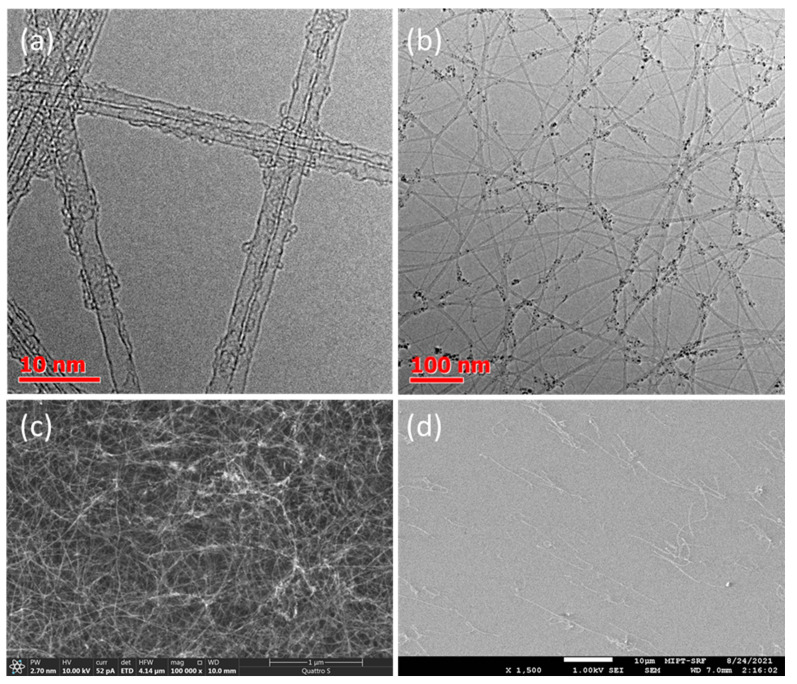
Typical TEM images of carbon nanotubes synthesized with ethylene (**a**,**b**); (**c**)—typical SEM image of SWCNT thin film; (**d**)—typical SEM image of individual carbon nanotubes deposited for length assessment.

Except the length distribution [51], the equivalent sheet resistance is also a function of the diameter distribution [21], defectiveness [21], metallic/semiconducting ratio [52], and bundle diameter [53] (as well as parameters maintained here: doping degree, ratio of metallic to semiconducting nanotubes, and film patterning). Figure 2c demonstrates that an increase in the ethylene concentration results in a rise in the I_G_/I_D_ ratio, indicating the improvement of the quality of the SWCNTs, which can be one of the reasons for the lower values of R_90_. At the same time, the SWCNT mean diameter estimated from Figure 2d based on the Kataura plot data did not depend on the carbon source concentration, unlike aerosol CVD methods based on ethylene/toluene hybrid sources [45,54]. It is worth noting that the presence of transitions between van Hove singularities, radial breathing modes in Raman spectra (Figure S1), and TEM images (Figure 3a,b) confirmed the formation of single-walled carbon nanotubes, while the obtained SWCNT films demonstrated random orientation (Figure 3c) of individual bundles (Figure 3d). 

It should be noted that, unlike some previous findings for floating catalyst CVD systems, we did not observe any synergy between the catalyst and carbon source [26,33,34], as the amount of ferrocene added just linearly increased the yield (Figure S2) without significant changes in the diameter distribution (Figure S3).

**Figure 4:**
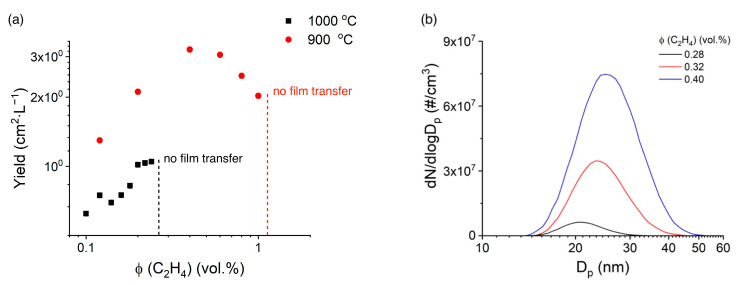
(**a**) Yield vs. ethylene concentration at different temperatures (ferrocene concentration 0.18 Pa). (**b**) DMA number size distributions of aerosol particles produced in the reactor under the C_2_H_4_/N_2_ environment. T = 1000 °C.

As mentioned before, comparing Figure 2a,b, we observed the optimal ethylene concentration for the SWCNT growth to be *ϕ* (C_2_H_4_) = 0.24 vol.% at 1000 °C; at that point, the yield achieved almost maximum value, while the resistance was almost at the lowest. However, a further increase in the ethylene concentration higher than *ϕ* (C_2_H_4_) = 0.24 vol.% deteriorated the quality of the SWCNT films, likely due to the formation of pyrolytic carbon, which made it impossible to dry-transfer the nanotube films from the filter. It is worth mentioning that only films containing high-quality and long SWCNTs can be easily transferred from a filter onto a secondary substrate [42]. At 900 °C, this boundary of the ethylene concentration was higher, i.e., *ϕ* (C_2_H_4_) = 1.0 vol.% (Figure 4a). Moreover, at high ethylene concentrations, DMA spectra (Figure 4b) showed the excessive formation of aerosol particles apart from carbon nanotubes. Based on these results, we can consider ethylene pyrolysis (i.e., non-catalytic decomposition) to be the reason for the limited nanotube growth.

Therefore, by varying the ethylene concentration, we achieved a catalytic ethylene decomposition threshold (*ϕ* (C_2_H_4_) = 0.40 vol.%), above which we observed gas-phase self-pyrolysis. To test this hypothesis, hydrogen was added to the synthesis of SWCNTs, which is known for its ability to hinder pyrolysis. It should be mentioned that the lower temperatures for carbon nanotube growth usually provide higher defectiveness [55] and dramatically decreased yield due to the kinetic limitations of the nanotube growth reaction [56,57,58].

### 3.2. Role of Hydrogen

**Figure 5:**
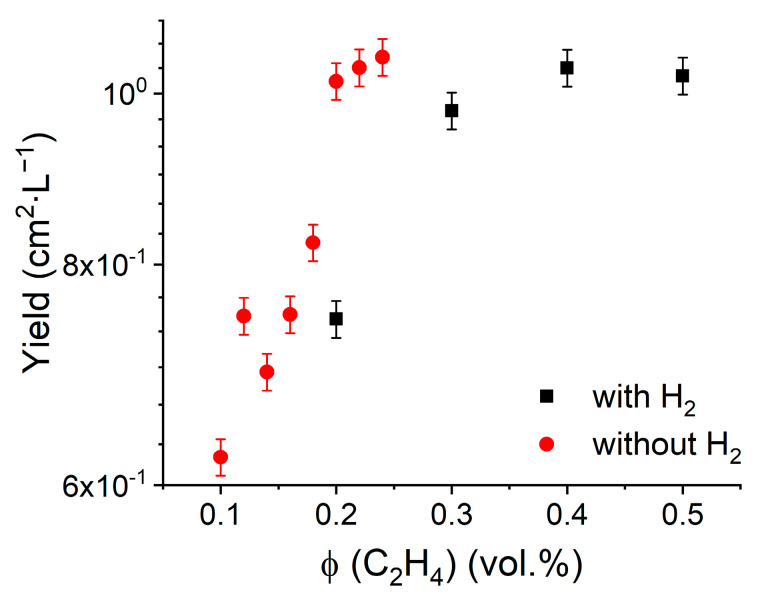
Yield as a function of ethylene concentration with (*ϕ* (H_2_) = 24 vol.%) and without hydrogen at T = 1000 °C.

As mentioned above, according to the classical studies of ethylene decomposition [36], hydrogen can suppress ethylene pyrolysis. After adding hydrogen with *ϕ* (H_2_) = 24 vol.% to the gas mixture, we were able to synthesize high-quality SWCNTs even at higher ethylene concentrations, up to *ϕ* (C_2_H_4_) = 0.5 vol.% (Figure 5). This phenomenon implies hydrogen expands the SWCNT growth window by suppressing pyrolysis.

To explore the effect, hydrogen concentration was varied in a wide range (0–30 vol%) at both temperatures (900 °C and 1000 °C) and fixed ethylene concentration (*ϕ* (C_2_H_4_) = 0.4 vol.%). Interestingly, the observed trends were unique for each temperature, revealing the complex effect of hydrogen. Moreover, not only the trends but also the characteristic concentrations of hydrogen drifted along.

**Figure 6:**
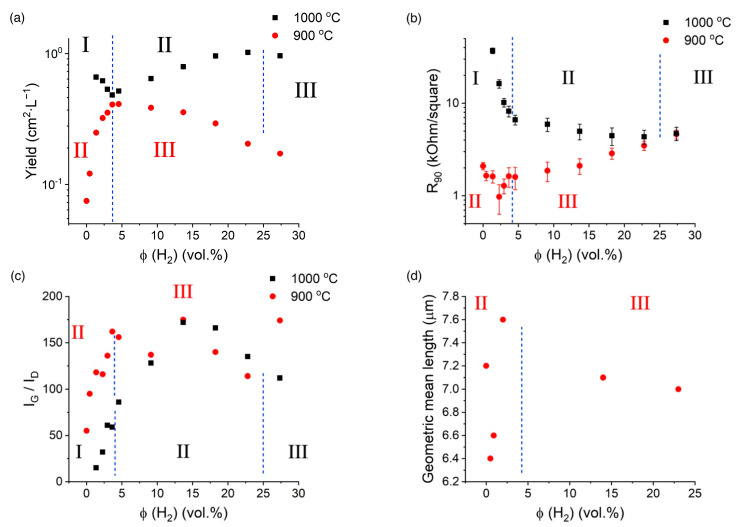
(**a**) Yield and (**b**) equivalent sheet resistance, and (**c**) Raman IG/ID ratio and (**d**) geometric mean length (Figure S5) vs. hydrogen concentration (T = 900 °C) as a function of hydrogen concentration at different temperatures at ethylene concentration *ϕ* (C2H4) = 0.4 vol.%.

At 900 °C, the increase in hydrogen amount enhanced yield, crystallinity, and length (Figure 6). However, at higher hydrogen content, we observed a reversed situation (except for the ratio of G to D mode). It is worth mentioning that we observed no changes in the SWCNT diameter throughout the whole range of hydrogen concentrations (Figure S4). Interestingly, the equivalent sheet resistance (R_90_) decreased with increased yield and length, proving that no significant changes in activation patterns were observed [21]. Indeed, the equivalent sheet resistance refers to the conductivity at the specific film thickness. While the increase in the length would intuitively decrease the film resistance, the increase in the activation would inevitably result in excessive bundling in the aerosol state increasing, thereby the film thickness without any significant contribution to sheet resistance. As R_90_ refers to a certain thickness, the increase in activation degree would most likely enhance the equivalent sheet resistance. As we did not observe such a situation here (Figure 5), and owing to constant diameter distribution (Figure S4), we can conclude that the hydrogen does not affect the activation pattern.

At 1000 °C, we observed three regimes: yield suppression with reduced R_90_ and enhanced quality at low concentrations; yield growth combined with an increase in quality and decrease in R_90_; the increase in sheet resistance and drop in the yield at high concentrations. While the second and third regimes closely resembled patterns observed at 900 °C, we explored an additional pattern at 1000 °C. The reduced R_90_ combined with suppression of the yield and increase in G to D mode ratio implied the removal of excessive carbon that does not take part in conductivity. Owing to the well-known hydrogen inhibition of pyrolysis [36], we most likely observed a suppression of ethylene pyrolysis [50]. 

Thus, based on the results (Figure 6), we propose the following three regimes for hydrogen effect on both temperatures:

- Pyrolysis suppression of ethylene (I regime) observed at *ϕ* (H_2_) < 3.6 vol.% at 1000 °C and not observed at 900 °C. The regime was characterized by a lower yield (Figure 6a), higher quality (Figure 6c), decreased equivalent sheet resistance of SWCNTs (Figure 6b), and increased length (Figure 6d).
- The surface cleaning/activation promotion (II regime) is the most optimal for the SWCNT synthesis (*ϕ* (H_2_) = 0.0–3.6 vol.% at 900 °C and *ϕ* (H_2_) = 4.6–23.0 vol.% at 1000 °C) and the catalyst surface cleaning resulting in increased SWCNT activation via the stable length (Figure 6d), defectiveness (Figure 6c), higher yield (Figure 6a), and slightly lower equivalent sheet resistance (Figure 6b).
- Reversible surface poisoning/SWCNT etching with hydrogen at high H_2_ concentrations (III regime: *ϕ* (H_2_) > 4.6 vol.% at 900 °C and *ϕ* (H_2_) > 27 vol.% at 1000 °C,) that manifests itself via low yield (Figure 6a), increased defectiveness (Figure 6c), and high R_90_ (Figure 6b).

## 4. Conclusions

To conclude, we studied the effect of hydrogen on the aerosol CVD synthesis of SWCNTs using ethylene as a carbon source. We observed hydrogen to inhibit ethylene pyrolysis, enlarging the growth zone of SWCNTs using ethylene as a carbon source. Moreover, the detailed study under different temperatures with a comprehensive set of methods allowed us to distinguish three distinct regimes for the hydrogen effect. The inhibition of ethylene pyrolysis (regime I: observed at T = 1000 °C only) allows removing polyaromatic carbons, improving the SWCNT performance even at the lowest hydrogen concentrations. Further increase in hydrogen presumably allows cleaning of the surface of the catalyst, enhancing the activation degree (regime II: *ϕ* (H_2_) = 0.0–3.6 vol.% at 900 °C and *ϕ* (H_2_) = 4.6–23.0 vol.% at 1000 °C). Nevertheless, after a certain threshold, hydrogen starts to suppress SWCNT growth (regime III) through presumable nanotube etching/surface poisoning affecting negatively the aerosol CVD process. We believe that such a detailed study will help to establish a comprehensive discussion on the role of hydrogen in the synthesis of SWCNTs from hydrocarbons.

## Data Availability

Not applicable.

## Supplementary Materials

The following supporting information can be downloaded at: https://www.mdpi.com/article/10.3390/nano13091504/s1, Figure S1. Raman spectra for SWCNT films obtained with different (a) ethylene (T = 1000 °C), (b) hydrogen (T = 900 °C) (c) hydrogen (T = 1000 °C) concentrations; Figure S2. (a) Deposition rate and (b) equivalent sheet resistance vs. ferrocene concentration at T_furnace_ = 1000 °C (black squares) and 900 °C (red circles), ∑_flowrate_ = 2500 sccm, C_2_H_4_ = 0.22 vol. %, Fe(C_5_H_5_)_2_ = x Pa; Figure S3. UV-Vis-NIR and Raman spectra of the obtained SWCNT films at different ferrocene concentrations. (a) UV-Vis-NIR spectra of SWCNT thin films with diameters calculated with the Kataura plot (b) Raman spectra and I_G_/I_D_ ratios in inset; Figure S4. Optical spectra for SWCNT films obtained with different hydrogen concentrations at (a) T = 900 °C, (b) T = 1000 °C; Figure S5. Length distribution of SWCNTs fitted by lognormal distribution curve with specified values of geometric mean length (GM), geometric standard deviation (σ), geometric mean (μ), and the statistical size of the sample (counts) for different hydrogen concentrations (T = 900 °C).

## References

[1] Monthioux M., Kuznetsov V.L. (2006). Who Should Be given the Credit for the Discovery of Carbon Nanotubes?. Carbon.

[2] Dresselhaus M.S., Dresselhaus G., Charlier J.C., Hernandez E. (2004). Electronic, Thermal and Mechanical Properties of Carbon Nanotubes. Philos. Trans. R. Soc. Lond. Ser. A Math. Phys. Eng. Sci..

[3] Fujisawa K., Kim H.J., Go S.H., Muramatsu H., Hayashi T., Endo M., Hirschmann T.C., Dresselhaus M.S., Kim Y.A., Araujo P.T. (2016). A Review of Double-Walled and Triple-Walled Carbon Nanotube Synthesis and Applications. Appl. Sci..

[4] Cheng H.M., Li F., Su G., Pan H.Y., He L.L., Sun X., Dresselhaus M.S. (1998). Large-Scale and Low-Cost Synthesis of Single-Walled Carbon Nanotubes by the Catalytic Pyrolysis of Hydrocarbons. Appl. Phys. Lett..

[5] Endo M., Hayashi T., Kim Y.A., Terrones M., Dresselhaus M.S. (2004). Applications of Carbon Nanotubes in the Twenty-First Century. Philos. Trans. R. Soc. A Math. Phys. Eng. Sci..

[6] Rao R., Pint C.L., Islam A.E., Weatherup R.S., Hofmann S., Meshot E.R., Wu F., Zhou C., Dee N., Amama P.B. (2018). Carbon Nanotubes and Related Nanomaterials: Critical Advances and Challenges for Synthesis toward Mainstream Commercial Applications. ACS Nano.

[7] Baughman R.H., Zakhidov A.A., De Heer W.A. (2002). Carbon Nanotubes—The Route Toward Applications. Science.

[8] Novikov I.V., Krasnikov D.V., Vorobei A.M., Zuev Y.I., Butt H.A., Fedorov F.S., Gusev S.A., Safonov A.A., Shulga E.V., Konev S.D. (2022). Multifunctional Elastic Nanocomposites with Extremely Low Concentrations of Single-Walled Carbon Nanotubes. ACS Appl. Mater. Interfaces.

[9] Butt H.A., Novikov I.V., Krasnikov D.V., Sulimov A.V., Pal A.K., Evlashin S.A., Vorobei A.M., Zuev Y.I., Ostrizhiniy D., Dzhurinskiy D. (2023). Binder-Free, Pre-Consolidated Single-Walled Carbon Nanotubes for Manufacturing Thermoset Nanocomposites. Carbon.

[10] Predtechenskiy M.R., Khasin A.A., Smirnov S.N., Bezrodny A.E., Bobrenok O.F., Dubov D.Y., Kosolapov A.G., Lyamysheva E.G., Muradyan V.E., Saik V.O. (2022). New Perspectives in SWCNT Applications: Tuball SWCNTs. Part 2. New Composite Materials through Augmentation with Tuball. Carbon Trends.

[11] Predtechenskiy M.R., Khasin A.A., Bezrodny A.E., Bobrenok O.F., Dubov D.Y., Muradyan V.E., Saik V.O., Smirnov S.N. (2022). New Perspectives in SWCNT Applications: Tuball SWCNTs.Part 1. Tuball by Itself—All You Need to Know about It. Carbon Trends.

[12] Iijima S., Ichihashi T. (1993). Single-Shell Carbon Nanotubes of 1-Nm Diameter. Nature.

[13] Li Y., Maruyama S., Li Y., Maruyama S. (2019). Single-Walled Carbon Nanotubes.

[14] Hirsch A., Backes C., Harris P.J.F. (2010). Carbon Nanotube Science. Synthesis, Properties and Applications.

[15] Ilatovskii D.A., Gilshtein E.P., Glukhova O.E., Nasibulin A.G. (2022). Transparent Conducting Films Based on Carbon Nanotubes: Rational Design toward the Theoretical Limit. Adv. Sci..

[16] Li Z., Kandel H.R., Dervishi E., Saini V., Biris A.S., Biris A.R., Lupu D. (2007). Does the Wall Number of Carbon Nanotubes Matter as Conductive Transparent Material?. Appl. Phys. Lett..

[17] Yang F., Wang M., Zhang D., Yang J., Zheng M., Li Y. (2020). Chirality Pure Carbon Nanotubes: Growth, Sorting, and Characterization. Chem. Rev..

[18] Zhang Q., Wei N., Laiho P., Kauppinen E.I. (2017). Recent Developments in Single-Walled Carbon Nanotube Thin Films Fabricated by Dry Floating Catalyst Chemical Vapor Deposition. Top Curr. Chem..

[19] Khabushev E.M., Krasnikov D.V., Zaremba O.T., Tsapenko A.P., Goldt A.E., Nasibulin A.G. (2019). Machine Learning for Tailoring Optoelectronic Properties of Single-Walled Carbon Nanotube Films. J. Phys. Chem. Lett..

[20] Weller L., Smail F.R., Elliott J.A., Windle A.H., Boies A.M., Hochgreb S. (2019). Mapping the Parameter Space for Direct-Spun Carbon Nanotube Aerogels. Carbon.

[21] Khabushev E.M., Krasnikov D.V., Kolodiazhnaia J.V., Bubis A.V., Nasibulin A.G. (2020). Structure-Dependent Performance of Single-Walled Carbon Nanotube Films in Transparent and Conductive Applications. Carbon.

[22] Hata K., Futaba D.N., Mizuno K., Namai T., Yumura M., Iijima S. (2004). Water-Assisted Highly Efficient Synthesis of Impurity-Free Single-Walled Carbon Nanotubes. Science.

[23] Yuan Y., Wei L., Jiang W., Goh K., Jiang R., Lau R., Chen Y. (2015). Sulfur-Induced Chirality Changes in Single-Walled Carbon Nanotube Synthesis by Ethanol Chemical Vapor Deposition on a Co/SiO_2_ Catalyst. J. Mater. Chem. A Mater..

[24] Balogh Z., Halasi G., Korbély B., Hernadi K. (2008). CVD-Synthesis of Multiwall Carbon Nanotubes over Potassium-Doped Supported Catalysts. Appl. Catal. A Gen..

[25] Nasibulin A.G., Brown D.P., Queipo P., Gonzalez D., Jiang H., Kauppinen E.I. (2006). An Essential Role of CO2 and H2O during Single-Walled CNT Synthesis from Carbon Monoxide. Chem. Phys. Lett..

[26] Guellati O., Janowska I., Bégin D., Guerioune M., Mekhalif Z., Delhalle J., Moldovan S., Ersen O., Pham-Huu C. (2012). Influence of Ethanol in the Presence of H 2 on the Catalytic Growth of Vertically Aligned Carbon Nanotubes. Appl. Catal. A Gen..

[27] Kuo D.H., Su M.Y. (2007). The Effects of Hydrogen and Temperature on the Growth and Microstructure of Carbon Nanotubes Obtained by the Fe(CO)_5_ Gas-Phase-Catalytic Chemical Vapor Deposition. Surf. Coat Technol..

[28] Zhang G., Mann D., Zhang L., Javey A., Li Y., Yenilmez E., Wang Q., Mcvittie J.P., Nishi Y., Gibbons J. (2005). Ultra-High-Yield Growth of Vertical Single-Walled Carbon Nanotubes: Hidden Roles of Hydrogen and Oxygen. Proc. Natl. Acad. Sci. USA.

[29] Franklin N.R., Li Y., Chen R.J., Javey A., Dai H. (2001). Patterned Growth of Single-Walled Carbon Nanotubes on Full 4-Inch Wafers. Appl. Phys. Lett..

[30] Rao F.B., Li T., Wang Y.L. (2008). Effect of Hydrogen on the Growth of Single-Walled Carbon Nanotubes by Thermal Chemical Vapor Deposition. Phys. E Low Dimens. Syst. Nanostruct..

[31] Chung U.C., Lee D.B., Jeong Y.U., Ha M.J., Chung W.S. (2005). Effect of H2 Gas on Carbon Nanotubes Synthesis. Mater. Sci. Forum.

[32] Li Y., Ji K., Duan Y., Meng G., Dai Z. (2017). Effect of Hydrogen Concentration on the Growth of Carbon Nanotube Arrays for Gecko-Inspired Adhesive Applications. Coatings.

[33] Ma Y., Dichiara A.B., He D., Zimmer L., Bai J. (2016). Control of Product Nature and Morphology by Adjusting the Hydrogen Content in a Continuous Chemical Vapor Deposition Process for Carbon Nanotube Synthesis. Carbon.

[34] Zhang H., Cao G., Wang Z., Yang Y., Shi Z., Gu Z. (2008). Influence of Ethylene and Hydrogen Flow Rates on the Wall Number, Crystallinity, and Length of Millimeter-Long Carbon Nanotube Array. J. Phys. Chem. C.

[35] Behr M.J., Gaulding E.A., Mkhoyan K.A., Aydil E.S. (2010). Effect of Hydrogen on Catalyst Nanoparticles in Carbon Nanotube Growth. J. Appl. Phys..

[36] Bokros J.C., Thrower P.A. (1969). Chemistry and Physics of Carbon.

[37] Tesner P.A., Rafalkes I.S. (1952). Dokl. Akad. Nauk. SSSR.

[38] Arefieva E.F., Snegireva T.D. (1978). Khimiya Tverd. Topl..

[39] Arefieva E.F., Snegireva T.D. (1978). Zhurnal Fiz. Khimii.

[40] Tesner P.A., Gorodetskii A.E., Snegireva T.D., Arefieva E.F. (1978). Dokl. Akad. Nauk. SSSR.

[41] Nasibulin A.G., Moisala A., Brown D.P., Jiang H., Kauppinen E.I. (2005). A Novel Aerosol Method for Single Walled Carbon Nanotube Synthesis. Chem. Phys. Lett..

[42] Kaskela A., Nasibulin A.G., Timmermans M.Y., Aitchison B., Papadimitratos A., Tian Y., Zhu Z., Jiang H., Brown D.P., Zakhidov A. (2010). Aerosol-Synthesized SWCNT Networks with Tunable Conductivity and Transparency by a Dry Transfer Technique. Nano Lett..

[43] Anoshkin I.V., Nasibulin A.G., Tian Y., Liu B., Jiang H., Kauppinen E.I. (2014). Hybrid Carbon Source for Single-Walled Carbon Nanotube Synthesis by Aerosol CVD Method. Carbon.

[44] Zhang Q., Huang J.Q., Zhao M.Q., Qian W.Z., Wei F. (2011). Carbon Nanotube Mass Production: Principles and Processes. ChemSusChem.

[45] Khabushev E.M., Krasnikov D.V., Goldt A.E., Fedorovskaya E.O., Tsapenko A.P., Zhang Q., Kauppinen E.I., Kallio T., Nasibulin A.G. (2022). Joint Effect of Ethylene and Toluene on Carbon Nanotube Growth. Carbon.

[46] Ermolaev G.A., Tsapenko A.P., Volkov V.S., Anisimov A.S., Gladush Y.G., Nasibulin A.G. (2020). Express Determination of Thickness and Dielectric Function of Single-Walled Carbon Nanotube Films. Appl. Phys. Lett..

[47] Dresselhaus M.S., Dresselhaus G., Saito R., Jorio A. (2005). Raman Spectroscopy of Carbon Nanotubes. Phys. Rep..

[48] Khabushev E.M., Kolodiazhnaia J.V., Krasnikov D.V., Nasibulin A.G. (2021). Activation of Catalyst Particles for Single-Walled Carbon Nanotube Synthesis. Chem. Eng. J..

[49] Hecht D., Hu L., Grüner G. (2006). Conductivity Scaling with Bundle Length and Diameter in Single Walled Carbon Nanotube Networks. Appl. Phys. Lett..

[50] Krasnikov D.V., Kuznetsov V.L., Romanenko A.I., Shmakov A.N. (2018). Side Reaction in Catalytic CVD Growth of Carbon Nanotubes: Surface Pyrolysis of a Hydrocarbon Precursor with the Formation of Lateral Carbon Deposits. Carbon.

[51] Novikov I.V., Khabushev E.M., Krasnikov D.V., Bubis A.V., Goldt A.E., Shandakov S.D., Nasibulin A.G. (2021). Residence time effect on single-walled carbon nanotube synthesis in an aerosol CVD reactor. Chem. Eng. J..

[52] Topinka M.A., Rowell M.W., Goldhaber-Gordon D., McGehee M.D., Hecht D.S., Gruner G. (2009). Charge Transport in Interpenetrating Networks of Semiconducting and Metallic Carbon Nanotubes. Nano Lett..

[53] Liao Y., Hussain A., Laiho P., Zhang Q., Tian Y., Wei N., Ding E.-X., Khan S.A., Nguyen N.N., Ahmad S. (2018). Tuning Geometry of SWCNTs by CO2 in Floating Catalyst CVD for High-Performance Transparent Conductive Films. Adv. Mater. Interfaces.

[54] Saito T., Ohshima S., Okazaki T., Ohmori S., Yumura M., Iijima S. (2008). Selective Diameter Control of Single-Walled Carbon Nanotubes in the Gas-Phase Synthesis. J. Nanosci. Nanotechnol..

[55] Krasnikov D.V., Bokova-Sirosh S.N., Tsendsuren T.-O., Romanenko A.I., Obraztsova E.D., Volodin V.A., Kuznetsov V.L. (2017). Influence of the Growth Temperature on the Defective Structure of the Multi-Walled Carbon Nanotubes. Phys. Status Solidi B.

[56] Nasibulin A.G., Queipo P., Shandakov S.D., Brown D.P., Jiang H., Pikhitsa P.V., Tolochko O.V., Kauppinen E.I. (2006). Studies on Mechanism of Single-Walled Carbon Nanotube Formation. J. Nanosci. Nanotechnol..

[57] Jourdain V., Bichara C. (2013). Current Understanding of the Growth of Carbon Nanotubes in Catalytic Chemical Vapour Deposition. Carbon.

[58] MacKenzie K.J., Dunens O.M., Harris A.T. (2010). An Updated Review of Synthesis Parameters and Growth Mechanisms for Carbon Nanotubes in Fluidized Beds. Ind. Eng. Chem. Res..

