# Supplementary material for: Role of Hydrogen in Ethylene-Based Synthesis of Single-Walled Carbon Nanotubes
Source: Nanomaterials (Basel). 2023 Apr 28;13(9):1504. doi: 10.3390/nano13091504 (PMC10180455; doi:10.3390/nano13091504)
Supplement: Supplementary file 1 [file nanomaterials-13-01504-s001.zip › nanomaterials-2322773-supplementary.pdf]

nanomaterials

Supplementary Materials

Role of Hydrogen in Ethylene-Based Synthesis of Single-Walled Carbon Nanotubes

Alisa R. Bogdanova [1], Dmitry V. Krasnikov [1], Eldar M. Khabushev [1], Javier A. Ramirez B. [1], Yakov E. Matyushkin [2] and Albert G. Nasibulin [1,*]

[1] Skolkovo Institute of Science and Technology, Nobelya Str. 3, Moscow 121205, Russia; d.krasnikov@skol.tech (D.V.K.); javier.ramirez@skoltech.ru (J.A.R.B.)
[2] Moscow Institute of Physics and Technology, Institute Lane 9, Dolgoprudny 141701, Russia
* Correspondence: a.nasibulin@skol.tech

The yield error was calculated based on the following reasoning. The error for the yield as an indirect measurement can be considered as a selective standard deviation according to the next equation:

$$S_{yield} = \sqrt{\left(\frac{\partial h}{\partial t}\right)^2\Bigg|_{t,T_{550}} S_t^2 + \left(\frac{\partial h}{\partial T_{550}}\right)^2\Bigg|_{t,T_{550}} S_{T_{550}}^2} = \sqrt{\left(-\frac{\log_{0.9} T_{550}}{t^2 Q}\frac{\pi d^2}{4}\right)^2\Bigg|_{t,T_{550}} S_t^2 + \left(\frac{1}{T_{550}\ln 0.9}\frac{\pi d^2}{4tQ}\right)^2\Bigg|_{t,T_{550}} S_{T_{550}}^2}$$

At the same time, $S_t = \sigma_{device}$, $\sigma_{device} \approx \frac{\Delta_{lim}}{3}$, $\Delta_{lim} = 0.001t + 1$

However, the selective standard deviation for the yield determination for samples obtained at the same conditions in a series of a repetitive measurements of n = 4 is calculated as:

$$S_{\bar{h}} = \sqrt{\frac{\sum_{i=1}^{n}\left(h_i - \bar{h}\right)^2}{n(n-1)}} = 9 \cdot 10^{-4}\ \mathrm{cm}^2 \cdot \mathrm{L}^{-1}$$

Thus, systematic error:

$$\sigma = \sqrt{S_h^2 + S_{\bar{h}}^2} = \left|S_h^2 \ll S_{\bar{h}}^2\right| = S_{\bar{h}} = 9 \cdot 10^{-4}\ \mathrm{cm}^2 \cdot \mathrm{L}^{-1}$$

Final relative standard deviation that was used for error bars:

$$C_v = \frac{S_{\bar{h}}}{\bar{h}} = 2.4\%$$

Indeed, it may appear that some figures have error bar while others do not. The reason is that the error is so small, that it is hard to see the error bars from the graph at first sight (Figure 3a, Figure 5a).

At the same time, the error for equivalent sheet resistance values was calculated according to the following formula:

$$S_{R_{90}} = \sqrt{\left(\frac{\partial R_{90}}{\partial R_s}\right)^2\Bigg|_{R_s,A_{550}} S_{R_s}^2 + \left(\frac{\partial R_{90}}{\partial A_{550}}\right)^2\Bigg|_{R_s,A_{550}} S_{A_{550}}^2} = \sqrt{\left(\frac{A_{550}}{\log(10/9)}\right)^2\Bigg|_{R_s,A_{550}} S_{R_s}^2 + \left(\frac{R_s}{\log(10/9)}\right)^2\Bigg|_{R_s,A_{550}} S_{A_{550}}^2}$$

where $S_{R_s} = \sqrt{\frac{\sum_{i=1}^{n}\left(R_{s_i} - \overline{R_s}\right)^2}{n(n-1)}}$

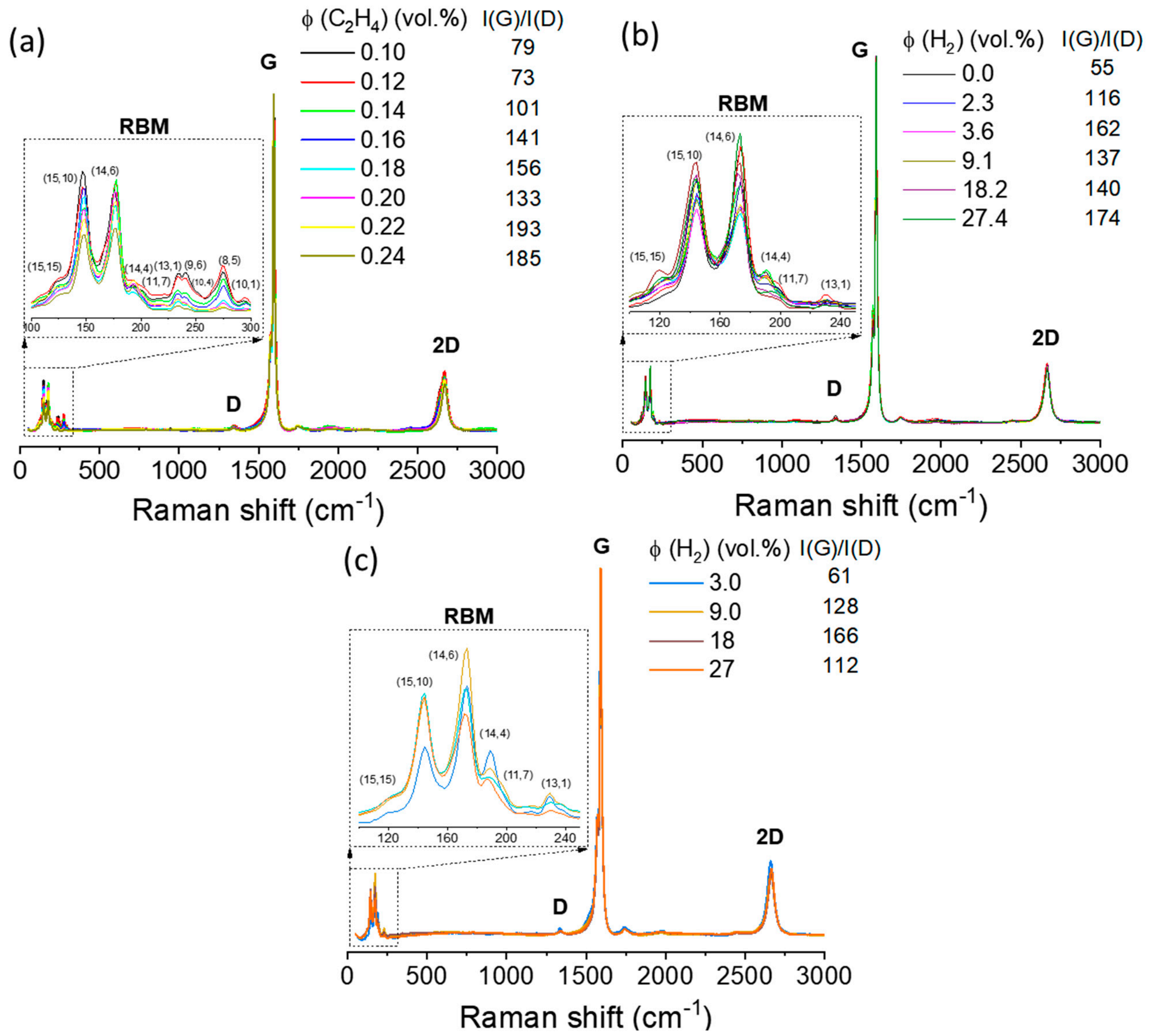

Figure S1. Raman spectra for SWCNT films obtained with different (**a**) ethylene (T = 1000 °C), (**b**) hydrogen (T = 900 °C) (**c**) hydrogen (T = 1000 °C) concentrations.

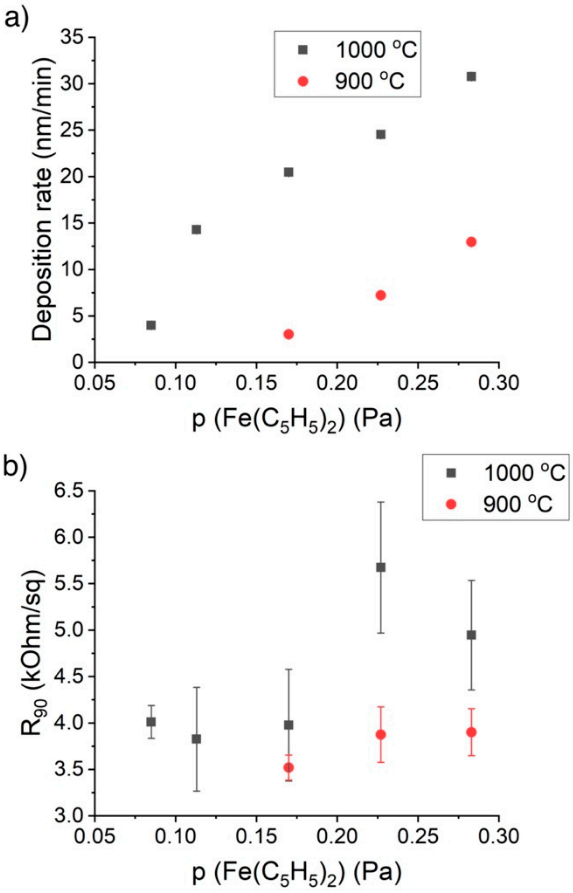

Figure S2. a) Deposition rate and **b**) equivalent sheet resistance vs. ferrocene concentration at $T_{furnace}$ = 1000 °C (black squares) and 900 °C (red circles), $\sum_{flowrate}$ = 2500 sccm, C_2H_4 = 0.22 vol. %, $Fe(C_5H_5)_2$ = x Pa.

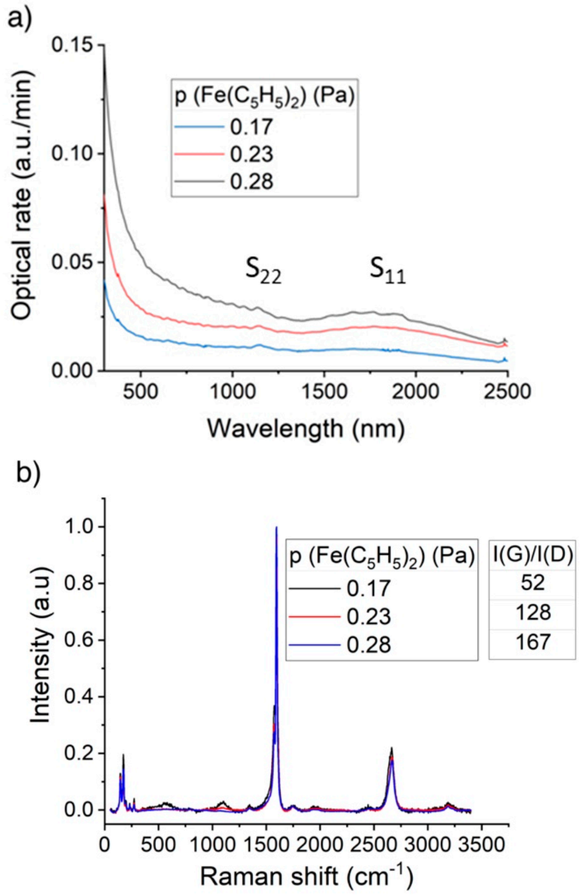

Figure S3. UV-Vis-NIR and Raman spectra of the obtained SWCNT films at different ferrocene concentrations. **a**) UV-Vis-NIR spectra of SWCNT thin films with diameters calculated with the Kataura plot **b**) Raman spectra and I_G/I_D ratios in inset.

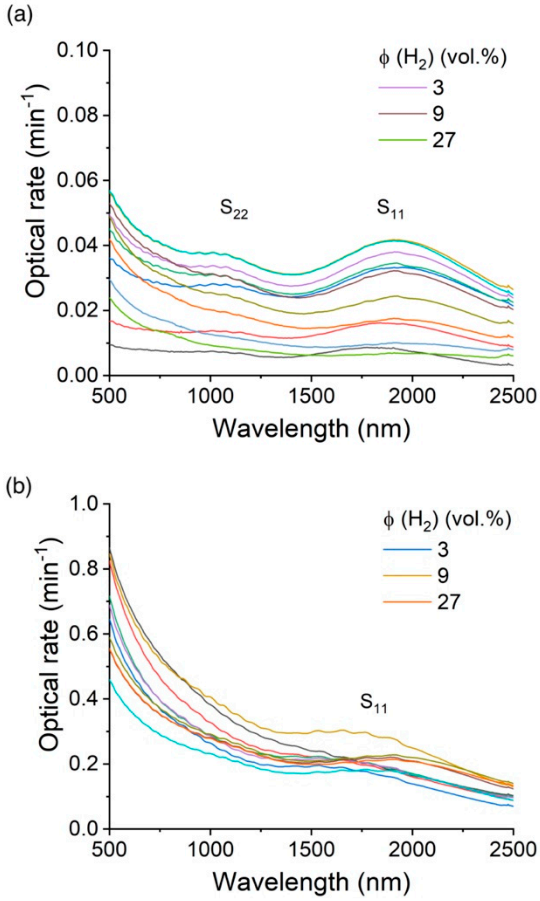

Figure S4. Optical spectra for SWCNT films obtained with different hydrogen concentrations at (**a**) T = 900 °C, (**b**) T = 1000 °C.

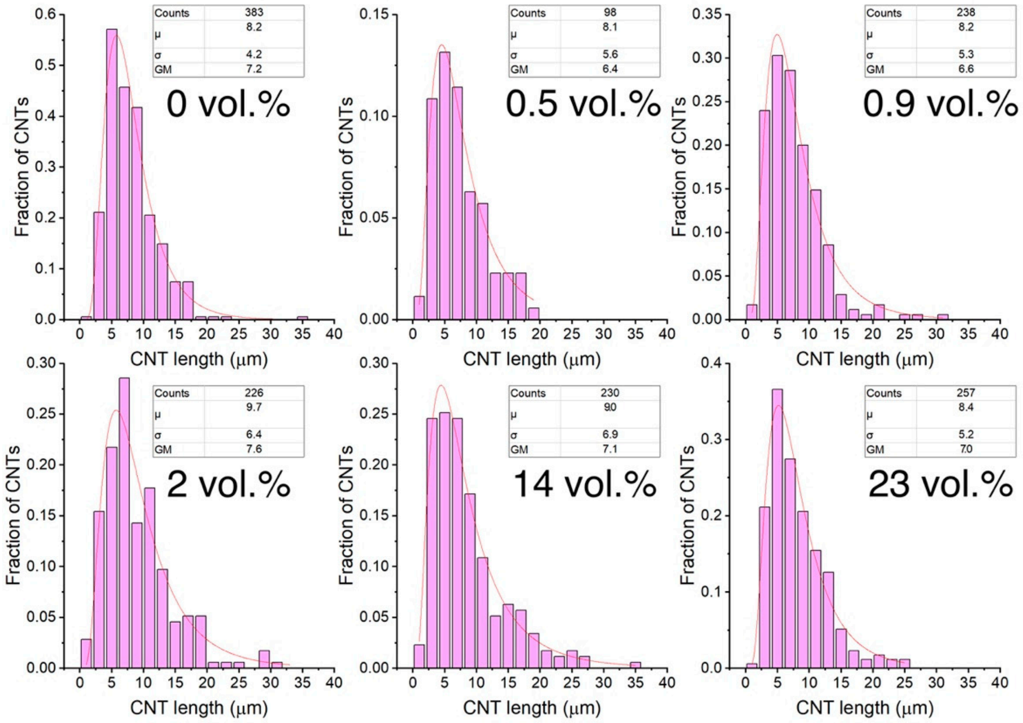

Figure S5. Length distribution of SWCNTs fitted by lognormal distribution curve with specified values of geometric mean length (GM), geometric standard deviation (σ), geometric mean (μ), and the statistical size of the sample (counts) for different hydrogen concentrations (T = 900 °C).
